# Cardiovascular progenitor cells cultured aboard the International Space Station exhibit altered developmental and functional properties

**DOI:** 10.1038/s41526-018-0048-x

**Published:** 2018-07-26

**Authors:** Jonathan Baio, Aida F. Martinez, Ivan Silva, Carla V. Hoehn, Stephanie Countryman, Leonard Bailey, Nahidh Hasaniya, Michael J. Pecaut, Mary Kearns-Jonker

**Affiliations:** 1 Department of Pathology and Human Anatomy, Loma Linda University, Loma Linda, CA USA; 2 BioServe Space Technologies, University of Colorado Boulder, Boulder, CO USA; 3 Department of Cardiovascular and Thoracic Surgery, Loma Linda University, Loma Linda, CA USA; 4 Division of Biomedical Engineering Sciences, Department of Basic Sciences, Loma Linda University, Loma Linda, CA USA

## Abstract

The heart and its cellular components are profoundly altered by missions to space and injury on Earth. Further research, however, is needed to characterize and address the molecular substrates of such changes. For this reason, neonatal and adult human cardiovascular progenitor cells (CPCs) were cultured aboard the International Space Station. Upon return to Earth, we measured changes in the expression of microRNAs and of genes related to mechanotransduction, cardiogenesis, cell cycling, DNA repair, and paracrine signaling. We additionally assessed endothelial-like tube formation, cell cycling, and migratory capacity of CPCs. Changes in microRNA expression were predicted to target extracellular matrix interactions and Hippo signaling in both neonatal and adult CPCs. Genes related to mechanotransduction (*YAP1, RHOA*) were downregulated, while the expression of cytoskeletal genes (*VIM*, *NES*, *DES*, *LMNB2*, *LMNA*), non-canonical Wnt ligands (*WNT5A*, *WNT9A*), and Wnt/calcium signaling molecules (*PLCG1*, *PRKCA*) was significantly elevated in neonatal CPCs. Increased mesendodermal gene expression along with decreased expression of mesodermal derivative markers (*TNNT2*, *VWF*, and *RUNX2*), reduced readiness to form endothelial-like tubes, and elevated expression of Bmp and Tbx genes, were observed in neonatal CPCs. Both neonatal and adult CPCs exhibited increased expression of DNA repair genes and paracrine factors, which was supported by enhanced migration. While spaceflight affects cytoskeletal organization and migration in neonatal and adult CPCs, only neonatal CPCs experienced increased expression of early developmental markers and an enhanced proliferative potential. Efforts to recapitulate the effects of spaceflight on Earth by regulating processes described herein may be a promising avenue for cardiac repair.

## Stem cells: Spaceflight changes nature of heart progenitors

Spaceflight alters the developmental status, proliferative potential and migratory ability of heart stem cells. Mary Kearns-Jonker from  Loma Linda University, California, USA, and colleagues characterized the molecular expression profiles of cardiovascular progenitor cells (CPCs) obtained from neonatal and adult patients and then cultured them aboard the International Space Station for 12 days. They found that spaceflight impacted the expression of microRNAs and genes related to cell cycling, DNA repair and important signaling events in both populations of CPCs, but there were some important differences. Of note, only neonatal CPCs showed enhanced potential for certain regenerative processes following spaceflight. Understanding the molecular basis for the beneficial effects induced in neonatal CPCs by spaceflight and mimicking these effects on Earth may boost the therapeutic efficacy of cell-based therapies. The findings also inform strategies to preserve the health of CPCs in astronauts as exploration of space expands in the future.

## Introduction

Changes to the cardiovascular system during spaceflight have prompted molecular biologists to understand the mechanisms governing cellular adaptation to culture aboard the International Space Station (ISS). In doing so, researchers have begun to identify therapeutic benefits, such as enhanced stemness, following culture in space and under conditions that approximate weightlessness on Earth. Therefore, increased understanding of the response of cardiovascular progenitor cells (CPCs) to spaceflight may not only benefit human health in space, but also provide insights into novel cardiovascular stem cell therapies that can be applied on Earth.

Recent research in our own laboratory has shown that neonatal human CPCs cultured under two-dimensional (2D) clinorotation, which approximates weightlessness by rotating the cell culture vessel about a horizontal axis, exhibit increased expression of markers of early cardiovascular development and pluripotency (i.e., *MESP1*, *T* (Brachyury), and *OCT4*) as well as broad changes to microRNA expression.^1^ The changes in microRNA expression experienced by CPCs in these experiments mirrored the expression of similar microRNAs in human embryonic stem cells (ESC) under normal conditions of early cardiovascular development, thereby suggesting that such culture conditions induce an epigenetic environment that favors an earlier development status. Such a state appears increasingly beneficial for cardiovascular repair. For example, the efficacy of multipotent stem cells expressing Isl1^+^ and SSEA-1^+^ in ameliorating the symptoms of severe heart failure was recently demonstrated in one patient.^2^ Furthermore, KEGG analysis indicated that the microRNAs downregulated in CPCs in these experiments target several signaling pathways, such as MAPK and Wnt, which are relevant to well-characterized repair mechanisms or cardiac stem cell proliferation mechanisms in the heart. For example, non-canonical Wnt ligands (i.e., Wnt2b, Wnt5a, and Wnt9a) have been shown to be associated with the injury response in neonatal rodent heart tissue.^3^ Therefore, our observations suggest that 2D clinorotation may foster a developmental state and signaling events that could enhance the use of CPCs for cardiovascular repair. This is supported by one recent study by Jha et al.^4^, which demonstrated that human induced pluripotent stem cells more readily differentiate into cardiomyocytes following three-dimensional (3D) (i.e., embryoid) culture under the influence of a random positioning machine. In the context of our previous findings, these experiments may highlight a potential phenomenon in which 2D clinorotation promotes an enhanced state of stemness that results in increased differentiation potential when the cells are returned to normal gravity conditions.

It is currently believed that small RhoGTPases act as transducers of the mechanical alterations that are observed under conditions of spaceflight.^5,6^ As actors in the planar cell polarity Wnt pathway, they have central roles in cytoskeletal remodeling. Along with the Hippo pathway and calcium signaling, small RhoGTPases represent one potential molecular substrate of gravity sensing. Notably, in the context of cardiogenesis, these processes are central to maintaining multipotency or directing differentiation.^7^ Despite the early reports of the effects of spaceflight on stem cells, much is unknown about the mechanisms by which CPCs respond to spaceflight or how these cell types develop enhanced stemness. Hence, we are not yet able to leverage the therapeutic potential of spaceflight here on Earth. While current clinical trials to stimulate repair in damaged heart tissue are promising,^8–10^ they are stymied by a failure of cells to engraft into the host tissue and by the use of progenitor types that are restricted in potency.^11^ Therefore, identifying the molecular events that promote enhanced stemness and regenerative potential in CPCs during spaceflight will benefit stem cell-based cardiac repair.

The successful use of autologous CPC sources to regenerate heart tissue in a predominately adult population requires additional understanding of the differences between adult and neonatal CPCs. Our laboratory has previously documented differences in age-dependent expression of microRNAs related to MAPK signaling, cytoskeleton regulation, adherens junction expression, and focal adhesion maintenance.^12^ The relationship between these processes and the age-dependent response of CPCs to spaceflight remain unknown, but they imply deficits in the intracellular transduction of environmental cues in the functional shortcomings of adult CPCs.

Therefore, in this study, clonal lines of neonatal and adult human CPCs were cultured aboard the ISS. We sought to identify changes in these CPCs in response to spaceflight that impact signaling, development, and stemness. We then characterized the divergent response of neonatal and adult CPCs to spaceflight. In doing so, we present components of the adaptive cellular response to culture aboard the ISS and their implications for enhancing the regenerative potential of neonatal and adult CPCs.

## Results

### CPCs exhibit markers of early cardiovascular development

**Fig. 1:**
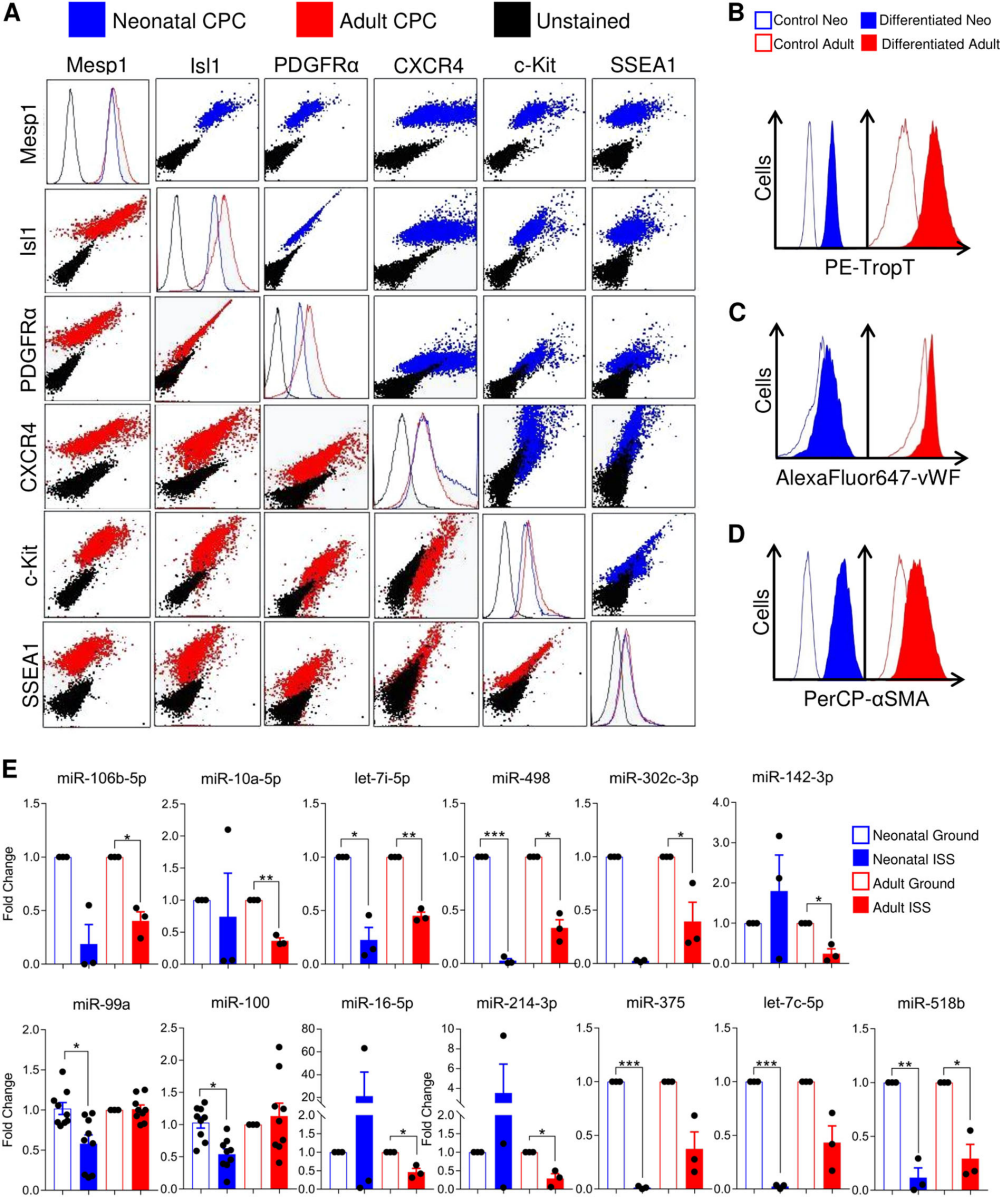
Spaceflight alters microRNA expression in neonatal and adult early CPCs. Neonatal (blue) and adult (red) CPC clones were screened for the co-expression of Isl1 and c-Kit, assessed for viability, and then ultimately selected for experiments based upon the co-expression of early developmental markers (Mesp1, PDGFRα, KDR, and SSEA1) along with the chemokine receptor CXCR4 **a**. Directed differentiation of these CPCs is able to induce cardiomyocytes **b**, endothelial cells **c**, and smooth muscle cells **d**. Neonatal and adult CPCs both exhibited significant alterations in microRNA expression following 12 days of culture aboard the ISS **e**. KEGG analysis (Table S1) indicated likely targeting of ECM interactions, membrane metabolism, and Hippo signaling by these microRNAs. *n* = 3 unique clones per age per group for all miRs, except miR-99a and miR100, which were measured in biological and technical triplicates. Data are reported as the mean ± SEM, *P < 0.05, **P < 0.01, ***P < 0.001

CPCs were clonally isolated, expanded, screened for the co-expression of Isl1 and c-Kit, assessed for viability, and ultimately selected for experiments based upon the expression of early developmental markers (Mesp1, PDGFRα, and SSEA1) along with the chemokine receptor CXCR4. Representative adult and neonatal clones are shown in Fig. 1a and Fig S1. Neonatal and adult CPCs are able to differentiate into cardiomyocytes (Fig. 1b), endothelial cells (Fig. 1c), and smooth muscle cells (Fig. 1d) when cultured using directed differentiation protocols (Fig. S2). Clonal lines of CPCs obtained from neonatal and adult patients were then flown aboard the ISS for 12 days and fixed in RNAprotect during orbit (Fig. S3).

### The spaceflight environment mediates changes to expression of microRNAs that target membrane synthesis and extracellular interactions

In an effort to understand the relevant signaling events by which CPCs adapt to culture aboard the ISS, we performed microarray analysis to measure broad alterations to transcriptional control. Among both neonatal and adult CPCs, 14 microRNAs exhibited significant alterations in levels of expression (Fig. 1e). KEGG analysis by age group (Table S1) indicated that fatty acid biosynthesis, extracellular matrix (ECM)–receptor interactions, and Hippo signaling were the most significantly affected targets of these microRNAs.

### Cytoskeletal maintenance is altered in neonatal, but not adult, CPCs after culture aboard the ISS

**Fig. 2:**
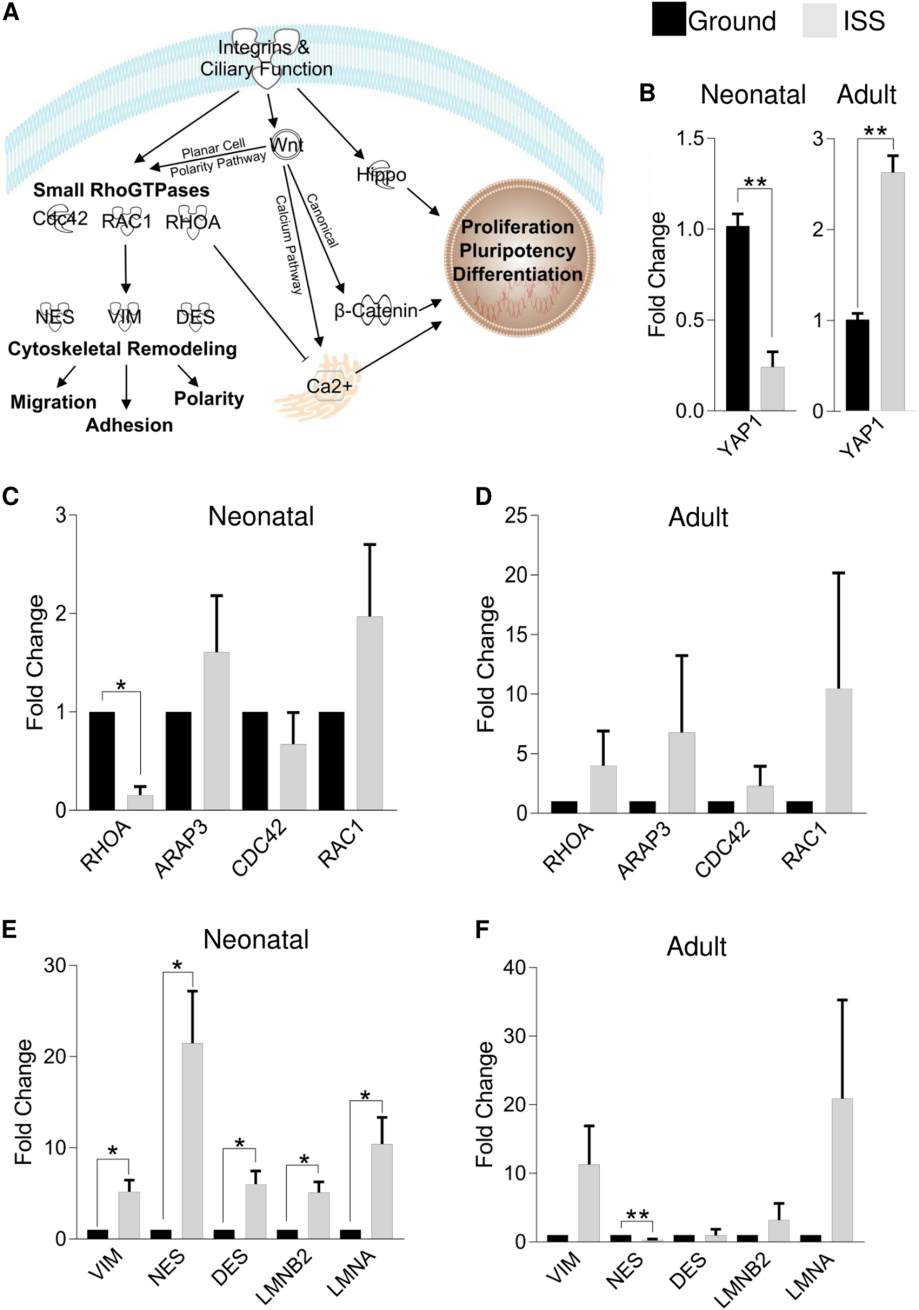
Spaceflight impacts expression of genes involved in mechanotransduction and cytoskeleton maintenance. Integrin and mechanical signaling impact Hippo activity and small RhoGTPases, which function along the Wnt planar cell polarity pathway **a**. *YAP1* expression was significantly reduced in neonatal CPCs and upregulated in adult CPCs **b**. *RHOA*, a small RhoGTPase, was expressed at significantly lower levels in neonatal **c**, but not adult **d**, CPCs. Accordingly, cytoskeletal gene expression was significantly upregulated in neonatal **e**, but not adult **f**, CPCs. *n* = 3 biological replicates for all gene expression data, except *YAP1* (*n* = 6 measures of three pooled clones). Data are reported as the mean ± SEM, *P < 0.05, **P < 0.01

Since the pathways targeted by the significantly dysregulated microRNAs affect cytoskeletal remodeling and mechanotransduction pathways (Fig. 2a), we sought to measure changes in the expression of *YAP1*, a gene involved in Hippo signaling; small RhoGTPases; and cytoskeletal molecules. *YAP1* was significantly downregulated in neonatal CPCs (0.24-fold, *P* < 0.01) and modestly upregulated in adult CPCs (2.6-fold, *P* < 0.01) (Fig. 2b). This prompted us to assess the mechanosensitive small RhoGTPases, which indicated that only *RHOA* was significantly downregulated in neonatal CPCs (0.15-fold, *P* < 0.05, Fig. 2c), which led us to assess the possible effects of spaceflight on cytoskeletal dysregulation. In doing so, we found that the cytoskeletal genes *VIM*, *NES*, *DES*, *LMNB2*, and *LMNA* were all significantly upregulated (5.2-, 22-, 6-, 5.1-, and 10-fold, respectively; *P* < 0.05) following spaceflight (Fig. 2e). Meanwhile, in adult CPCs, only *NES* expression decreased significantly (0.30-fold, *P* < 0.01) (Fig. 2d, f).

### Transcripts for non-canonical/Ca^2+^ signaling in neonatal CPCs are elevated during spaceflight

**Fig. 3:**
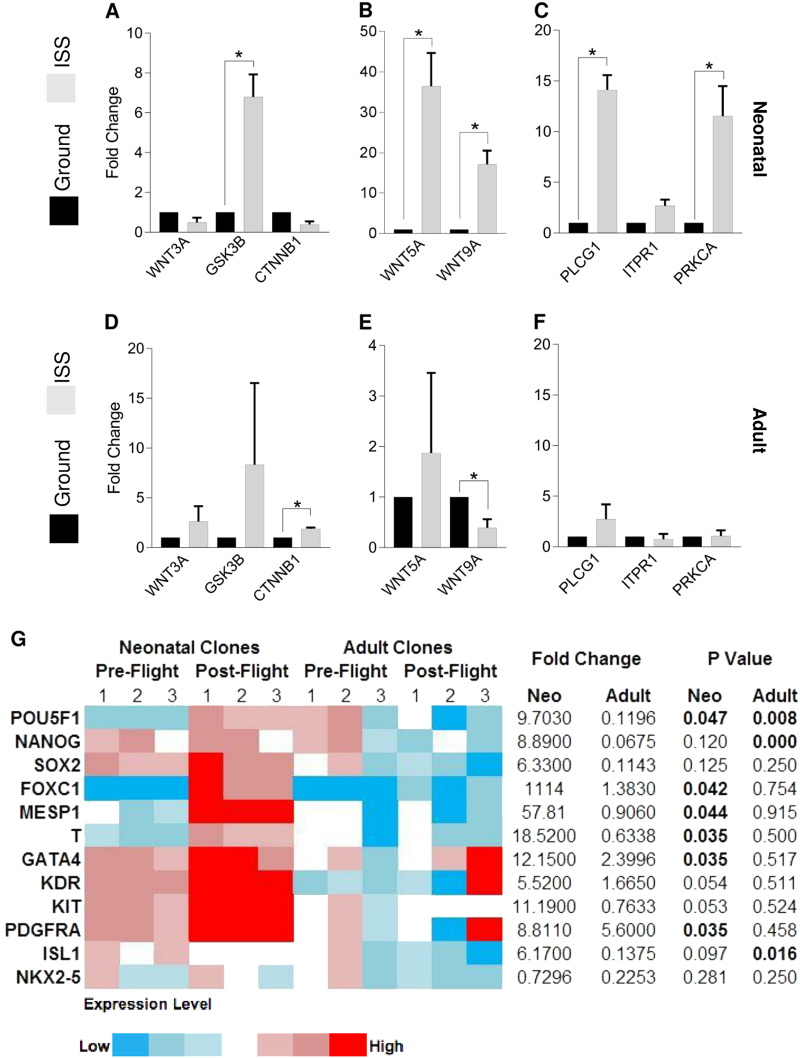
Genes involved in the non-canonical Wnt/Ca^2+^ pathway are expressed at higher levels along with mesendodermal markers in neonatal CPCs. Canonical Wnt signaling genes were measured in neonatal **a** and adult **d** CPCs along with non-canonical Wnt ligands **b**, **e**. Genes involved in the non-canonical Wnt/calcium pathway were elevated in neonatal **c**, but not adult **f**, CPCs. Markers of development were measured in neonatal and adult CPCs, which indicated elevation of early mesodermal markers (i.e., *FOXC1*, *MESP1*, and *T*) in the former and no changes in the latter population **g**. *n* = 3 unique clones per group. Data are reported as the mean ± SEM, *P < 0.05

Given the role of small RhoGTPases in non-canonical, planar cell polarity Wnt signaling and the impact of RhoA on regulating calcium signaling,^13^ we sought to assess the expression of genes involved in the canonical and calcium Wnt pathways. (Fig. 3a–f). We observed significantly increased expression of *GSK3B* (6.8-fold, *P* < 0.05) in neonatal CPCs (Fig. 3a) and significantly increased expression of *CTNNB1* (1.9-fold, *P* < 0.05) in adult CPCs (Fig. 3d). Since Gsk3β sequesters β-catenin for degradation in the cytoplasm, thereby suggesting suppression of canonical Wnt signaling in neonatal CPCs and the promotion of canonical Wnt signaling in adult CPCs, we measured non-canonical Wnt ligand expression and that of genes in the Wnt/Ca^2+^ pathway. We measured significantly increased *WNT5A* and *WNT9A* expression (36-fold and 17-fold, respectively; *P* < 0.05) in neonatal CPCs (Fig. 3b) and significantly decreased *WNT9A* expression (0.39-fold, *P* < 0.05) in adult CPCs (Fig. 3e). This was supported by significantly increased expression of *PLCG1* and *PRKCA* (14-fold and 12-fold, respectively; *P* < 0.05) and a general increase in *ITPR1* expression in neonatal CPCs (Fig. 3c), but not adult CPCs (Fig. 3f).

### Neonatal CPCs exhibit increased expression of genes involved in pre-mesodermal development

Calcium and Wnt signaling have central roles in cardiogenesis. For this reason, we sought to assess the transcription of markers of various stages of cardiac development. In doing so, we observed that the expression of genes involved in embryonic stem cell self-renewal (*POU5F1*, *NANOG*, *SOX2*), mesodermal specification (*FOXC1*, *MESP1*, *T*), and early cardiogenesis (*GATA4*, *KIT*, *PDGFRA*, *ISL1*, *KDR)* increased, while *NKX2-5* expression decreased, in neonatal CPCs (Fig. 3g). In this population, the expression of genes involved in early mesodermal specification were the most significantly elevated. Conversely, adult CPCs generally exhibited decreased or unchanged expression of genes involved in early developmental processes (Fig. 3g).

### Expression of BMP4 and TBXs 3, 5, and 18 is elevated under ISS culture in neonatal CPCs

**Fig. 4:**
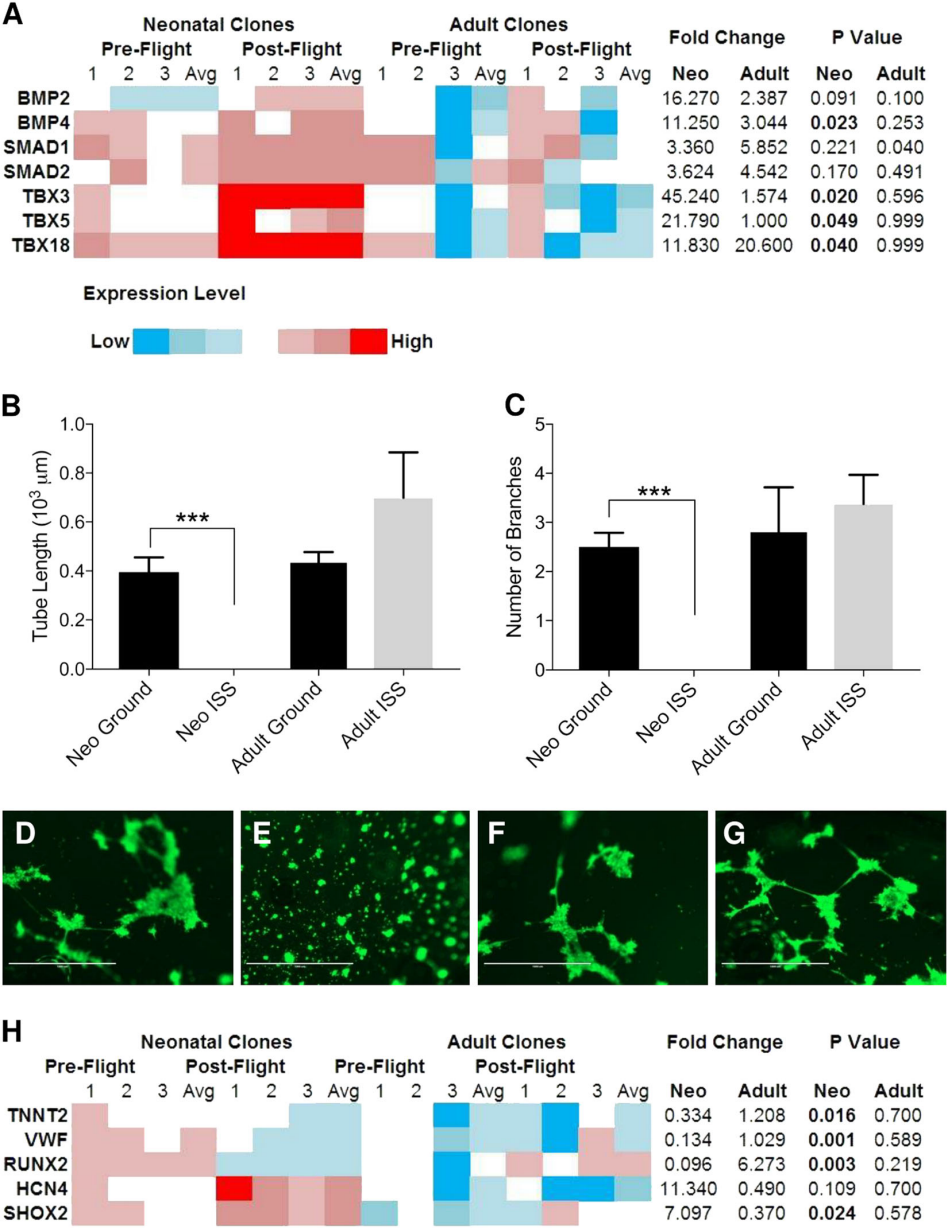
Spaceflight reduces the propensity for neonatal CPCs to readily form endothelial-like tubes and express markers of terminal mesodermal derivatives. The expression of members of the BMP, Smad, and TBX families of genes was measured **a** and found to be elevated significantly in neonatal CPCs and modestly in adult CPCs. Following culture in biocells on the ground or the ISS for 30 days, CPCs were placed in endothelial growth media and incubated on Matrigel for 7 h to facilitate endothelial-like tube formation. The length of endothelial-like tubes formed **b** and the number of branches **c** were quantified using ImageJ for neonatal ground **d** and ISS **e** CPCs as well as for adult ground **f** and ISS **g** CPCs (ruler = 1000 µm). Changes in gene expression relevant to mesodermal derivatives were measured in CPCs cultured aboard the ISS for 12 days, indicating decreased expression of cardiomyocyte, endothelial, and osteogenic markers and increased expression of sinoatrial nodal markers **h**. *n* = 3 unique clones per group for gene expression data; *n* = 3 measurements of four pooled clones per group for migration assay data. Data are reported as the mean ± SEM, ***P < 0.001

These modifications to the expression of genes involved in cardiogenesis motivated us to measure the changes in the expression of genes in the Bmp, Smad, and Tbx families, which have important roles in pre- and early mesodermal development. We measured elevated expression of *BMP4*, *TBX3*, *TBX5*, and *TBX18*, and a general increase in *BMP2* expression in neonatal CPCs (Fig. 4a). In adult CPCs, *SMAD1* exhibited a significant increase in expression, while *SMAD2* and *TBX18* also trended towards an increase in expression (Fig. 4a). These findings indicate that the transcripts of genes involved in early pre-cardiac mesoderm development are upregulated by spaceflight in neonatal CPCs.

### Spaceflight reduces terminal mesodermal derivative marker expression and endothelial-like tube formation in neonatal CPCs

Given the elevated expression of early to pre-mesodermal genes in neonatal, but not adult, CPCs, we sought to measure whether spaceflight impacts the expression of CPC and other mesodermal derivative markers. First, following 30 days in orbit, CPCs were returned to Earth and assayed for endothelial-like tube formation using a widely accepted assay to assess the potency of progenitor cells with the capacity to form tube-like structures.^14,15^ However, endothelial-specific markers were not measured on space-flown CPCs in this assay. Unlike adult CPCs, neonatal CPCs exhibited a significantly reduced ability to form endothelial-like tubes (Fig. 4b, c). Representative images of endothelial-like tube formation of neonatal ground (Fig. 4d) and ISS (Fig. 4e) as well as adult ground (Fig. 4f) and ISS (Fig. 4g) samples reflect this age-dependent response of CPCs to spaceflight. This impact of ISS culture on endothelial-like tube formation ability led us to measure the expression of markers of other derivatives: cardiomyocyte (*TNNT2*), endothelial (*VWF*), and osteoblast (*RUNX2*). These markers were all significantly reduced in neonatal, but not adult, CPCs after 12 days in orbit. Since spaceflight induces pre-cardiac mesodermal marker expression, we sought to assess whether markers of the sinoatrial node, which may represent embryonic myocardium maintained in its primitive state,^16^ were modified. Indeed, the sinoatrial nodal markers *HCN4* and *SHOX2* exhibited elevated expression in neonatal CPCs and reduced expression in adult CPCs (Fig. 4h). Taken together, these findings indicate that spaceflight induces a pre-cardiac mesoderm gene expression profile in neonatal CPCs.

### ISS-cultured neonatal CPCs proliferate more rapidly than adult or ground-control CPCs

**Fig. 5:**
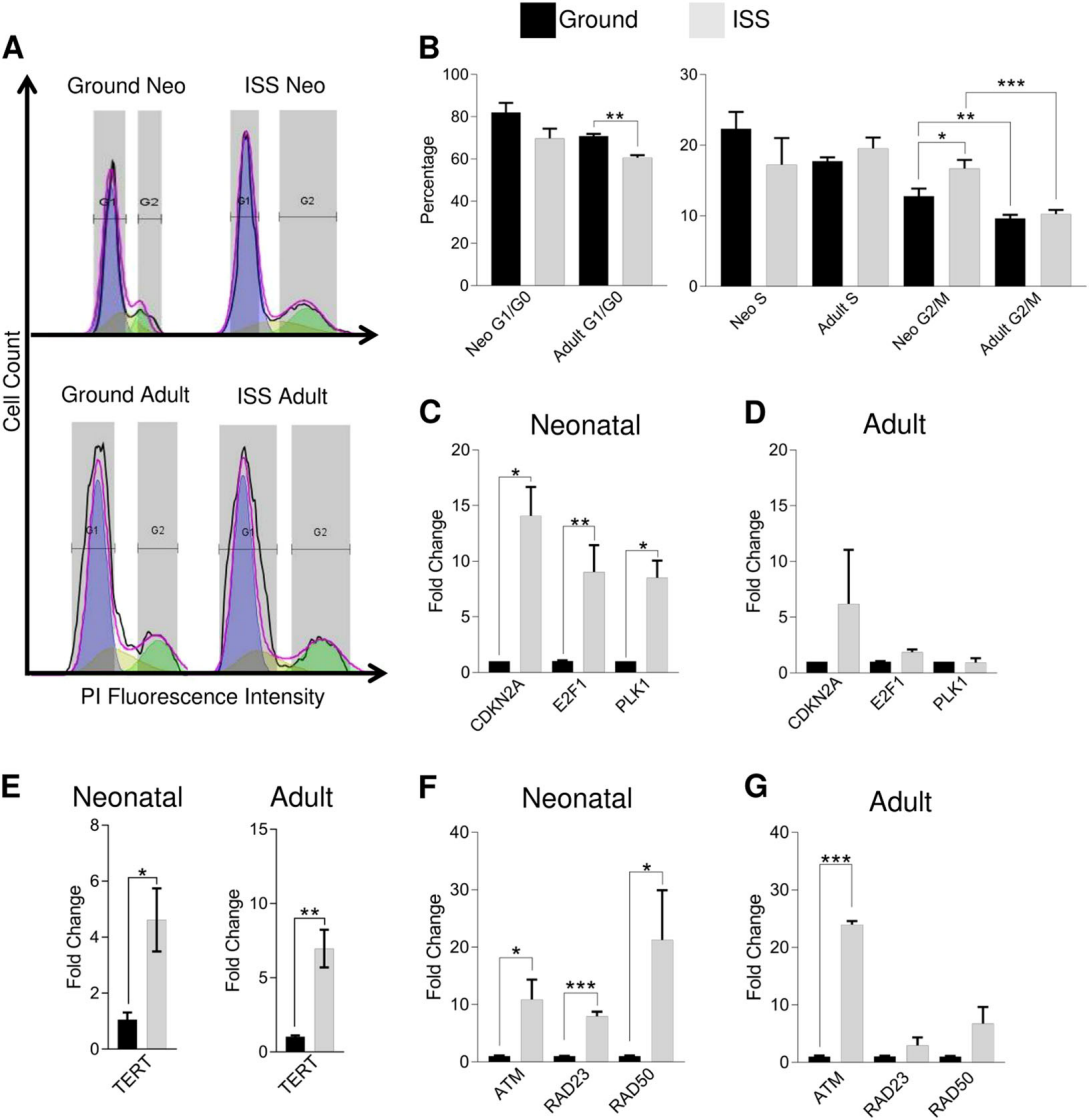
Cell cycling and proliferation are enhanced following spaceflight in neonatal CPCs. Following 30 days of culture aboard the ISS, CPCs were fixed, stained with propidium iodide, and measured using flow cytometry. The Dean–Jett–Fox model was then applied to the histogram of propidium iodide fluorescence intensity for ground-cultured and ISS-cultured CPCs **a**. Upon analysis **b**, adult CPCs exhibited a significant decrease in the percentage of cells in the G1/G0 and neonatal CPCs exhibited a significant increase in the percentage of cells in the G2/M phase. This was supported by increased expression of *CDKN2A*, *E2F1*, and *PLK1*, which function to regulate G1/S arrest, overcome G1/S arrests, and promote G2/M progression, respectively, in neonatal **c**, but not adult **d**, CPCs. Furthermore, increased telomerase activity, as indicated by enhanced *TERT* expression **e**, was observed in both groups. In addition to enhanced proliferation, DNA repair gene expression was induced in neonatal CPCs, as indicated by increased levels of *ATM*, *RAD23*, and *RAD50* **f**. Adult CPCs generally exhibited increased DNA repair genes; however, only *ATM* expression was significantly increased **g**. *n* = 3 measurements of four pooled clones per group for cell cycling analysis; *n* = 3 unique clones per group for all gene expression data, except for *E2F1*, *TERT*, *ATM*, *RAD23*, and *RAD50* (*n* = 9, three biological samples each measured in triplicate). Data are reported as the mean ± SEM, *P < 0.05, **P < 0.01, ***P < 0.001

Given the change in developmental status of the cell, we sought to assess whether such changes would affect the proliferative potential of space-flown CPCs; hence, cell cycling was assessed using flow cytometry. The Dean–Jett–Fox model was fitted to fluorescence intensity curves generated for propidium iodide-stained neonatal and adult CPCs (Fig. 5a). Adult CPCs exhibited a significant decrease in the percentage of cells in the G1/G0 phase (ISS vs Ground: 71% vs 61%, P < 0.01) and neonatal CPCs exhibited a significant increase in the percentage of cells in the G2/M phase (ISS vs Ground: 14% vs 17%, P = 0.05) (Fig. 5b). Neonatal CPCs were more frequently in the G2/M phase both on the ground and following culture aboard the ISS. Interestingly, neonatal CPCs exhibited increases in *CDKN2A* (14-fold, *P* < 0.05), *E2F1* (9-fold, *P* = 0.01), and *PLK1* (8.5-fold, *P* < 0.05) (Fig. 5c). These modulators of cell cycling were not significantly altered in adult CPCs (Fig. 5d). Furthermore, increased telomerase activity, as indicated by enhanced *TERT* expression (Fig. 5e), was observed in neonatal (6.8-fold, *P* < 0.05) and adult (21-fold, *P* < 0.01) CPCs. Regardless of age, CPCs demonstrate enhanced proliferative potential aboard the ISS.

### DNA repair transcripts increased following spaceflight

The broad alterations experienced by CPCs and the possibility of exposure to radiation in the extraterrestrial environment prompted us to consider possible cellular stress response mechanisms, including the expression of DNA repair gene programs. In doing so, we measured increased expression of genes involved in DNA repair, as indicated by elevated levels of *ATM* (11-fold, *P* < 0.05), *RAD23* (7.9-fold, *P* < 0.001), and *RAD50* (21-fold, *P* < 0.05) (Fig. 5f). Adult CPCs generally exhibited increased expression of DNA repair genes (Fig. 5g); however, only *ATM* (24-fold, *P* = 0.001) expression was significantly elevated.

### Stress response, but not apoptosis, genes are induced in CPCs

Given the broad induction of transcripts associated with DNA repair, we sought to characterize the impact of other stressors that could influence cellular function during spaceflight. Neonatal CPCs exhibited a 25-fold increase (*P* < 0.05) in *NOTCH1* expression (Fig S4A, B), which confers protection as part of the adaptive cardiac stress response.^17^ In considering stress associated with spaceflight (Fig S4C, D),^18,19,20^ we found that *HSP70* was elevated in both neonatal (12-fold, *P* < 0.01) and adult (20-fold, *P* < 0.05) CPCs, while *TXNIP*, *TP53INP*, and *HMOX1* were all induced only in adult CPCs (21-fold, *P* < 0.01; 2.2-fold, *P* < 0.05; 3.2-fold, *P* < 0.001; respectively). Ultimately, neither adult nor neonatal CPCs exhibited an induction of transcripts associated with the apoptosis pathway (Fig S4E, F). Indeed, neither proliferation nor viability was reduced in either age group (Fig S5A, B).

### Adult and neonatal CPCs both exhibit a greater migratory capacity after ISS culture

**Fig. 6:**
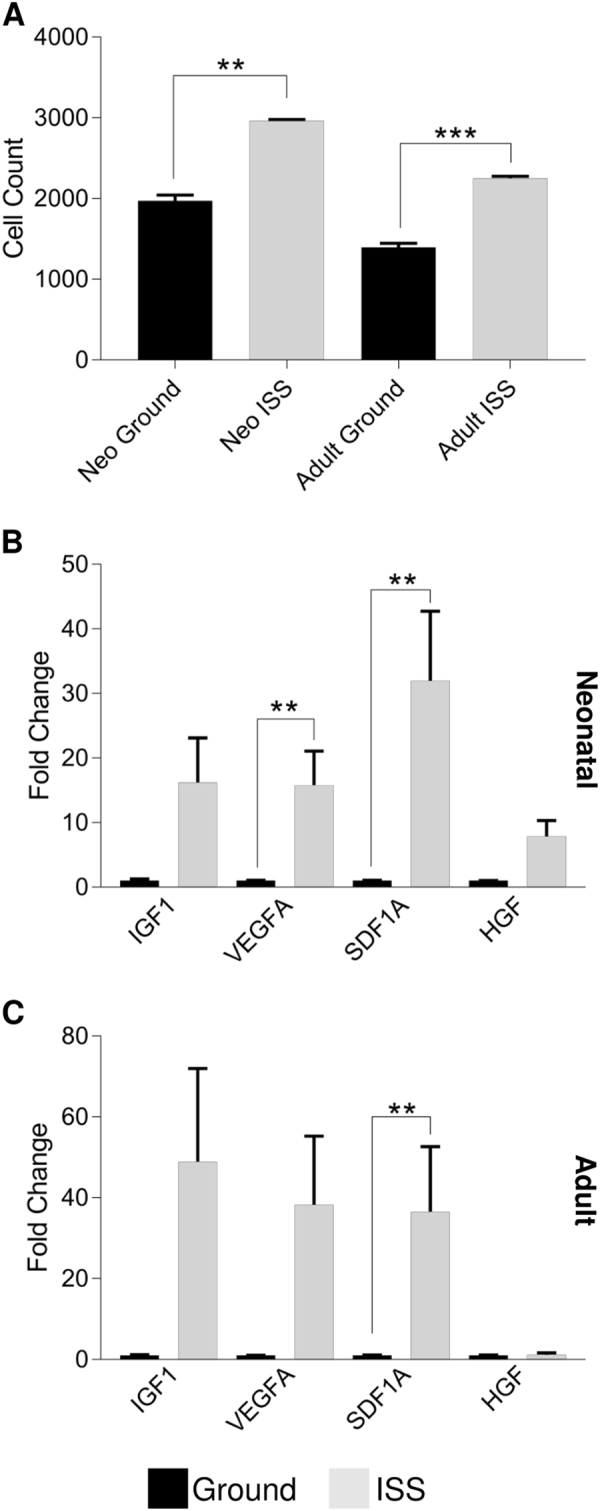
CPCs have enhanced migratory capacity following spaceflight. Both neonatal and adult CPCs that were cultured aboard the ISS migrated at significantly higher levels in response to SDF-1α stimulation **a**. Similarly, *SDF1A* expression was elevated in both neonatal and adult CPCs following exposure to low Earth orbit **b**, **c**. *VEGFA* expression was also significantly elevated in neonatal CPCs **b**, while most other paracrine and growth factors generally increased regardless of age. *n* = 5 measurements of four pooled clones per group for migration assay; *n* = 3 unique clones each measured in triplicate for gene expression experiments. Data are reported as the mean ± SEM, **P < 0.01, ***P < 0.001

In addition to changes in the regulation of differentiation and proliferation, cytoskeletal alterations were anticipated to occur in response to spaceflight. For this reason, we measured the migratory capacity of ISS-cultured CPCs using a Transwell migration assay. Both neonatal and adult CPCs that were cultured aboard the ISS migrated at significantly higher levels in response to SDF-1α stimulation (neonate (ISS vs Ground): 2963 ± 15 cells vs 1972 ± 69 cells, *P* < 0.01; adult (ISS v Ground): 2247 ± 28 cells vs 1394 ± 52 cells, *P* < 0.001; Fig. 6a). Similarly, *SDF1A* expression was elevated in both neonatal (32-fold, *P* < 0.01, Fig. 6b) and adult (37-fold, *P* < 0.01, Fig. 6c) CPCs following exposure to low Earth orbit. *VEGFA* expression was also significantly elevated in neonatal CPCs (16-fold, *P* < 0.01). Thus, CPCs, regardless of age, demonstrate enhanced migratory capacity aboard the ISS.

## Discussion

In this study, we found that spaceflight exerts broad effects on the developmental status, proliferative potential, and migratory ability of CPCs, some of which occurred in an age-dependent manner. In particular, neonatal CPCs exhibited increased expression of early developmental markers, enhanced proliferative potential, and increased migratory capacity following culture aboard the ISS. Meanwhile, adult CPCs experienced little change in the expression of genes indicative of their developmental state, but exhibited changes to migratory capacity. These findings suggest that broad cytoskeletal modifications resulting from reduced mechanotransduction impart improved migratory and adhesion capabilities (Fig. 2a) regardless of age, but that neonatal CPCs are able to propagate or experience additional intracellular signaling events (e.g., calcium signaling) that modify their developmental status. As we have shown elsewhere, one important difference between adult and neonatal CPCs is their epigenetic (i.e., microRNA) environment.^12^

Several microRNAs were observed to be differentially regulated in response to spaceflight in neonatal and adult CPCs. Interestingly, the predicted targets of these microRNAs included activators and repressors of several pathways. A detailed inspection of the putative targets under each pathway impacted by the microRNAs identified in this study revealed the targeting of genes relating to Wnt signaling (*FZD3*, *FZD5*, *FZD7*, *FZD8*), cytoskeletal regulation (*ACTB*, *ACTG1*, *LAMC3*), cardiovascular development (*TGFBR1*, *BMP2*, *BMP5*, *SMAD7*), integrin function (*ITGB4*, *ITGAV*), extracellular proteins (*COL4A2*, *COL5A1*, *COL1A1*, *COL1A2*, *COL4A1)*, and Hippo signaling (*TEAD1*, *YAP1*, *LATS1*). Interestingly, miR-16, whose expression was generally elevated in neonatal CPCs and repressed in adult CPCs, directly targets *YAP1*, which could account, at least in part, for the age-dependent response of CPCs to spaceflight. However, since KEGG analysis only identifies broad categories of genes (i.e., functional categories) that are likely targets of the microRNAs under study, further experiments are warranted to identify the cumulative impact of these microRNAs on signaling in space-flown CPCs.

These results motivated us to assess the overall impact of spaceflight on *YAP1*, an effector of Hippo signaling that functions with TAZ to regulate transcription. Activation of Hippo kinases, such as through a loss of microRNA-mediated repression or through reduced mechanical signaling, results in elevated levels of phosphorylated Yap1 and Taz. This prevents translocation of these transcriptional activators to the nucleus. Similarly, the application of 2D clinorotation to bone mesenchymal stem cells prevented TAZ translocation to the nucleus, thereby preventing the expression of *RUNX2* and inhibiting osteogenesis.^21^ Similarly, the decreased expression of *YAP1* and *RUNX2* observed in this experiment indicate that spaceflight reduces mechanotransduction in neonatal CPCs.

In addition to broadly targeting the Hippo signaling pathway and ECM interactions, select microRNAs that were modified by culture aboard the ISS are known to be critically involved in cardiac development. For example, miR-142-3p is involved in targeting genes involved in early cardiac development, such as *TBX5*.^22^ Meanwhile, miR-302, which targets the type II BMP receptor to regulate early cardiogenic events, is repressed by Bmp4 in a Smad-dependent manner.^23^ Interestingly, we observed both reduced expression of miR-302c and elevated expression of *BMP4* in CPCs that were cultured aboard the ISS. Moreover, studies have demonstrated a direct mechanistic role of certain microRNAs in cardiac regeneration. As neonatal mice age, their cardiomyocytes lose their proliferative potential in a manner that is concomitant with increased let-7 family expression,^24^ thereby indicating a relationship between let-7 over-expression and reduced proliferative potential. Indeed, let-7c along with miR-99/100, which were significantly downregulated in neonatal CPCs, are also critically downregulated during cardiomyocyte proliferation in a zebrafish model after ventricle amputation.^25^ As part of the amputation injury response, zebrafish cardiomyocytes near the site of injury undergo sarcomeric structure disassembly as a form of limited de-differentiation to prepare myocytes for self-renewal. *PLK1*, which was upregulated in CPCs cultured aboard the ISS, is involved in this process.^26^ Interestingly, these changes to the cytoarchitecture of cardiomyocytes and subsequent limited de-differentiation appears to resemble the effects of 2D clinorotation^12,27^ and spaceflight on CPCs.

Our laboratory has previously measured changes in microRNA expression in neonatal CPCs induced by 2D clinorotation.^12^ In comparing those findings to the microRNA changes induced by culture aboard the ISS, we found miR-99a and -100 as well as members of the let-7 family, all of which are implicated in differentiation and developmental regulation, exhibit significantly reduced expression under both conditions. Additionally, we have previously compared the effects of spaceflight and 2D clinorotation on the induction of signaling in neonatal CCPs and observed similar activation of calcium and Akt signaling under both conditions. Similarly, both clinorotation and spaceflight induced a gene expression profile that was consistent with an earlier stage of cardiovascular development, and that mechanical signaling processes were involved, at least in part, in mediating these effects. When considered together, these findings indicate that both processes readily alter the developmental properties of neonatal CPCs, possibly through reduced mechanotransduction. Therefore, the recapitulation of spaceflight-induced signaling events using clinorotation may represent an avenue by which ongoing studies can be conducted. This comparative study also allows for the separation of effects due to cell culture stress from those due to mechanical unloading since 2D clinorotation, which utilized cell cultures that were maintained under ideal conditions, elicited a similar effect as spaceflight.^27^

Alterations to microRNA expression as well as changes to ECM interactions in ISS-cultured CPCs highlight the significance of the environment in directing differentiation. For example, a single population of mesenchymal stem cells can express neurogenic, myogenic, or osteogenic transcription factors when cultured on soft, moderately stiff, or stiff matrices, respectively.^28^ In our experiments, we observed elevated expression of mesendodermal-stage developmental markers in neonatal CPCs. It is likely that ECM interactions prompted cytoskeleton remodeling while modifying intracellular signaling pathways such that proliferation and stemness were enhanced. Similar experiments in different cell types involving clinorotation or a low Earth orbit cell culture system have demonstrated that such systems modify the differentiation potential of stem cells. Mouse ESCs flown as embryoid bodies during the Space Tissue Loss Experiment exhibited a decrease in the expression of markers of terminal germ layer derivatives while maintaining expression of stem cell renewal markers.^29^ However, upon return to Earth, these mouse ESCs differentiated more readily into colonies of contracting cardiomyocytes. Mesenchymal stem cells cultured under conditions of 2D or 3D clinorotation exhibit a reduction in the expression of markers of cartilage and osteoblast formation,^30,31^ while also demonstrating enhanced proliferation potential and the maintenance or enhancement of stemness under such clinorotation.^30,32^

In addition to the expression of genes that are indicative of an earlier stage of development, neonatal CPCs exhibited both a reduced capacity to readily form endothelial-like tubes, as evidenced by the absence of any tube-like structures being formed that are indicative of endothelial-potential,^14,15^ and decreased expression of mesodermal derivative markers (i.e., cardiomyocte, endothelium, and osteoblast) in response to spaceflight. Notably, the increased expression of sinoatrial nodal markers may reflect the emerging hypothesis that this unique functional cell type develops from myocardium that retains its primitive phenotype.^16^ Accordingly, the concomitant increased expression of sinoatrial nodal genes and decreased expression of other mesodermal derivatives indicates that spaceflight promotes the modest de-differentiation of neonatal CPCs. Additionally, the elevated expression of Tbx proteins, particularly, Tbx3, overlaps with previous reports in the regulation of pre-mesoderm (i.e., mesendoderm) gene expression.^33^

This concomitant induction of mesendodermal markers and Tbx gene expression reflects the potential therapeutic value of spaceflight for cardiac repair. The critical role of the Tbx family in cardiac development has been well documented^34^ and its application to cellular therapies for cardiac repair is beginning to be explored. For example, SSEA-1^+^ early cardiovascular progenitors expressing *T*, *TBX5*, and *TBX18*, were shown to engraft into non-human primate myocardium following infarction.^35^ Meanwhile, *Tbx5* and *Tbx18* are expressed in highly migratory and proliferative proepicardial cell lineages.^36,37^ These studies have demonstrated an increasingly appreciated observation that Tbx expression is related to the engraftment and function of CPCs following transplantation.

Beyond directing developmental events via alterations in intracellular signaling, disrupted cytoskeletal organization impacts migration. In other models, cytoskeletal re-organization induces the expression of *CXCR4*, the SDF-1α chemokine receptor.^38^ As seen in our experiments, both neonatal and adult CPCs exhibited elevated expression of *SDF1A*, which correlates to the age-independent increase in migratory capacity of CPCs following spaceflight.

Interestingly, the age-dependent changes in developmental status following culture aboard the ISS suggest fundamental differences in adult CPCs. While KEGG analysis demonstrated that functionally similar pathways are likely affected in both adult and neonatal CPCs, only the latter population experienced significant changes to the expression of cytoskeletal genes and early developmental markers. This trend was supported by the significantly increased cell cycle and proliferation patterns observed only in neonatal CPCs. Broad differences have already been reported to exist between adult and neonatal CPCs, including at the genetic/epigenetic^12^ and proteomic levels.^39^ In particular, the secretome of adult CPCs was found to exhibit a greater number of proteins related to anti-proliferation, pro-apoptosis, and senescence. Meanwhile, the secretome of neonatal CPCs exhibited higher levels of telomerases as well as proteins related to cell cycle, proliferation, and anti-apoptosis.^39^ Similarly, we observed age-disparate responses to spaceflight in nearly all of these categories. In the context of this experiment, microRNA dysregulation may have exerted a greater effect on neonatal CPCs given their relatively higher levels of expression of genes related to these processes. Nevertheless, the prospective use of CPCs in autologous cardiac stem cell therapies in a predominately adult population requires that these functional differences be elucidated.

Although the ISS represents a truly unique opportunity to conduct life science research in space, there are several limitations that do not exist in the well-controlled laboratory environments on Earth. In addition to the stress associated with rocket launch, the limited availability of astronauts once aboard the ISS impacts the otherwise routine maintenance of cell cultures. For this reason, future research should consider the influence of various spaceflight-related stressors, such as sheer and vibrational stressors, on stem cell processes under normal gravity conditions. For example, we have shown that 2D clinorotation on Earth, which was performed in a carefully controlled laboratory setting, impacted the developmental properties of CPCs in a manner that was similar to spaceflight.^27^

As humans prepare to expand our presence in space, it is imperative to deepen our understanding of the nature of cellular adaptation to spaceflight so that we may develop mechanisms by which these molecular changes in cardiac cell types can be countered. Meanwhile, on Earth, we can further explore the therapeutic potential of these adaptations for cardiac repair.

## Methods

### Isolation and culture of early CPCs

CPCs were isolated from cardiac tissue of neonatal (1 day–1 month) or adult (57–72 year old) human patients, as previously described.^27^ The methods were performed in accordance with relevant guidelines and regulations and were approved by the Institutional Review Board of Loma Linda University for the use of discarded tissue without identifiable private information under a waiver of informed consent. Thus, informed consent was not required since the discarded tissue was not collected specifically for this study, and the tissue was obtained with no identifiable patient-specific information. Briefly, atrial tissue was cut into small clumps (approximately 1.0 mm^3^) then enzymatically digested using collagenase (Roche, Indianapolis, IN) at a working concentration of 1 mg/mL. The resulting solution was then passed through a 40-µm cell strainer. Cells were cloned in a 96-well plate by limiting dilution to a final concentration of 0.8 cells per well to create populations for expansion. Cells were then screened for the co-expression of Isl1 and c-Kit. Clonal CPC cultures were selected for other markers of early cardiac development (KDR and PDGFRA) and then supplemented with growth media comprised of 10% fetal bovine serum (Thermo Scientific, Waltham, MA), 100 µg/mL penicillin–streptomycin (Life Technologies, Carlsbad, CA), 1% minimum essential medium non-essential amino acids solution (Life Technologies, Carlsbad, CA), and 22% endothelial cell growth media (Lonza, Basel, Switzerland) in Medium 199 (Life Technologies, Carlsbad, CA). Mycoplasma contamination was tested using the MycoAlert Mycoplasma Detection Kit (Lonza, Basel, Switzerland) following the manufacturer’s protocol.

### Flow cytometry

Progenitor cell populations were fluorescently labeled with antibodies, as recommended by their respective manufacturers. Briefly, for developmental marker detection, CPCs were incubated with Viobility 405/520 before being washed in 1 × PBS (Life Technologies, Grand Island, NY) containing 0.5% BSA (Research Products International Corp, Mt. Prospect, IL) and 2 mM EDTA (Sigma Aldrich, St. Louis, MO). Then, CPCs were stained for cardiac-related stem cell surface markers (PDGFRα, CXCR4, c-Kit, and SSEA1). CPCs were then fixed in 4% PFA (Sigma Aldrich, St. Louis, MO), permeabilized in 0.1% Tween-20 (Sigma Aldrich, St. Louis, MO), blocked in 0.6 M glycine (Sigma Aldrich, St. Louis, MO) solution containing 10% BSA (Research Products International Corp, Mt. Prospect, IL), and stained for intracellular, cardiac-related stem cell surface markers (Isl1 and Mesp1). For directed differentiation assays, cells were stained for TropT after being fixed in 4% PFA (Sigma Aldrich, St. Louis, MO), permeabilized in 0.1% Tween-20 (Sigma Aldrich, St. Louis, MO) or directly for vWF or SMA. Stained CPCs were then analyzed using a MACSQuant® analyzer (Miltenyi Biotec, Auburn, CA). Quantification of data was performed using FlowJo software version 10 (Ashland, OR). Compensation was performed using UltraComp eBeads (Life Technologies, Grand Island, NY) following the manufacturer’s protocol. Briefly, one drop of compensation beads were incubated with each antibody individually for 30 min at 4 °C and then detected using the same MACSQuant® analyzer just prior to experiments requiring compensation. FlowJo was then used to generate a compensation matrix, which was applied to all experimental data. Information concerning antibodies, isotype controls, and reagents used in antibody labeling is provided in Table S2.

### Directed differentiation of CPCs

To validate the ability of neonatal and adult CPCs to differentiate into cells of the cardiovascular lineage, directed differentiation assays were performed, as described in Le et al.^40^ and Smits et al.^41^ In brief, to generate cardiomyocytes, CPCs were cultured in growth media (see above) until 85% confluent. CPC growth media was then replaced with differentiation media (47% IMDM, 47% Ham’s F12 with GlutaMAX, 2% penicillin–streptomycin, 2% horse serum, 1% minimum essential medium non-essential amino acids solution, and 1% insulin-transferring selenium (all reagents from Life Technologies, Carlsbad, CA) containing 5 µM 5-Azacytidine (Sigma, St. Louis, MO)), which was refreshed daily for 3 days. Then, 1 ng/mL TGF-β (R&D Systems, Minneapolis, MN) and 0.1 mM ascorbic acid (Acros Organics, Geel, Belgium) in fresh differentiation media was added routinely until day 14, at which time cells were stained for TropT or placed in TRIzol® reagent (Life Technologies, Carlsbad, CA) for gene expression analysis. To generate endothelium, CPCs were cultured in growth media (see above) until 85% confluent, at which time the growth media was replaced with endothelial cell growth media (Lonza, Basel, Switzerland) containing 25 ng/mL Bmp4 (R&D Systems, Minneapolis, MN) and 50 ng/mL Vegf (R&D Systems, Minneapolis, MN) for 2 days. The media was then replaced with endothelial cell growth media (Lonza, Basel, Switzerland) containing 50 ng/mL Vegf and cultured for 4 days. Then, cells were regularly fed endothelial cell growth media (Lonza, Basel, Switzerland) until day 14, at which time cells were stained for vWF, placed in TRIzol® reagent (Life Technologies, Carlsbad, CA) for gene expression analysis, or seeded onto Matrigel for endothelial-like tube formation assay analysis, as described below. Finally, to generate smooth muscle cells, CPCs were cultured in growth media (see above) until 85% confluent, at which time the growth media was replaced with DMEM (Life Technologies, Carlsbad, CA) containing 4.5 g/L d-glucose, 2% fetal bovine serum (Thermo Scientific, Waltham, MA), and 50 ng/mL PDGF-BB (R&D Systems, Minneapolis, MN). This media was regularly replaced until day 14, at which time cells were stained for smooth muscle actin or placed in TRIzol® reagent (Life Technologies, Carlsbad, CA) for gene expression analysis.

### CPC culture aboard the ISS

Neonatal and adult CPCs were seeded into Biocells (BioServe Space Technologies, Boulder, CO) at two seeding densities (7500 cells or 5000 cells) and loaded into self-contained environments containing 5% CO_2_ and 95% air. The seeding conditions were carefully titrated on Earth to identify the most optimal conditions under which CPCs could be cultured over the course of the experiment. These conditions were then tested during an Experiment Verification Test, which was performed with BioServe Space Technologies ahead of the mission launch. They were then flown aboard SpaceX CRS-11 to the US National Lab on the ISS where they were placed in an incubator containing 5% CO_2_ and 95% air. Fresh media was provided every 4 to 5 days while aboard the ISS (Fig S3). At 12 days, the Biocells that were seeded with 7500 cells were flooded with RNAProtect (Qiagen, Valencia, CA) and stored at −80 °C. At 30 days, the Biocells seeded with 5000 cells were fed and returned to Earth. Clone-matched and passage-matched ground controls were fed and treated in parallel with the feeding schedule and activities performed by astronauts aboard the ISS.

### Post-flight sample processing

Upon landing and retrieval of the payload, live cells were trypsinized, counted, and used to assess cell cycle, migration, and endothelial-like tube formation, as well as to prepare protein lysates. Biocells containing cells fixed and frozen in RNAprotect were thawed at room temperature. The RNAprotect was removed and centrifuged at 10,000×*g* at 4 °C for 10 min. Biocells were disassembled and the culture membranes were then rinsed with TRIzol® reagent (Life Technologies, Carlsbad, CA). RNA was purified from the RNAprotect samples using the RNeasy Mini Kit (Qiagen, Valencia, CA), per the manufacturer’s instructions, while total RNA was purified from TRIzol® reagent using isopropanol-based and ethanol-based precipitation. cDNA was generated and RT-PCR was performed as described below.

### RT^2^ microRNA profiler array and miRNA RT-PCR

MicroRNA array profiling was performed as previously described.^1^ Briefly, the miScript II RT Kit (Qiagen, Valencia, CA) was used to convert 500 ng of total RNA into cDNA, which was diluted, added to miScript SYBR Green PCR master mix and 10× universal primer mix (Qiagen, Valencia, CA), and run on human development and differentiation miScript plates (MIHS-103ZA; Qiagen, Valencia, CA). Fold changes were determined for each clone using the Qiagen Data Analysis Center (http://www.qiagen.com/us/shop/genes-and-pathways/data-analysis-center-overview-page/) using all available housekeeping genes. Since this analysis center performs only a two-tailed Student’s *t*-test to calculate *P*-values, all fold changes for individual clones were exported to Prism and analyzed, as described below. Individual primers for SNORD96a, SNORD72, hsa-miR-100-5p, and hsa-miR-99a-5p (SABiosciences, Valencia CA) were also used (Table S2). The average *C*_*t*_ value of SNORD96a and SNORD72 was used as a housekeeping control. The PCR conditions for the microRNA arrays and individual microRNA assays were: 95 °C for 15 min and 40 cycles of 94 °C for 15 s, 55 °C for 60 s, and 70 °C for 30 s. Differences in fold changes between ground-cultured and ISS-cultured CPCs were then analyzed as described in the statistics sub-section.

### MicroRNA targeting prediction

KEGG analysis was performed using DIANA mirPath version 3 and TarBase v7.0^42^ for microRNAs that were found to be significantly altered in neonatal and adult CPCs. Results were merged by pathway union after controlling for error rate due to multiple comparisons via selecting the “correct for FDR” feature. After removing categories related to cancer, neurological diseases, and viruses/pathogenesis, the KEGG categories whose *P*-values were <0.05 were reported.

### RT^2^ profiler array

We used custom array plates (CLAH22469A; Qiagen, Valencia, CA) per the manufacturer’s instructions to measure gene expression changes in ISS-cultured adult and neonatal CPCs that were relevant to Wnt, ERK, BMP/Smad, and Notch signaling; cytoskeletal maintenance; calcium handling; apoptosis and cell cycle; cardiac development and stemness; and regeneration. Briefly, 2 µg of RNA was reverse transcribed into cDNA, as described above, and then thoroughly mixed with 2×RT^2^ SYBR Green Mastermix and RNase-free and DNase-free water before being loaded into the profiler array plate. Samples were amplified in the iCycler iQ™5 PCR Thermal Cycler (Bio-Rad, Hercules, CA) using a protocol of 95 °C for 10 min and 40 cycles of 95 °C for 15 s and 60 °C for 1 min. Fold changes were determined for each clone individually using the Qiagen Data Analysis Center (http://www.qiagen.com/us/shop/genes-and-pathways/data-analysis-center-overview-page/) using *GAPDH* and *ACTB* (β-actin) as housekeeping genes. Since this analysis center performs only a two-tailed Student’s *t*-test to calculate *P*-values, all fold changes for individual clones were exported to Prism and analyzed, as described below.

### Quantitative RT-PCR

Changes in the expression of select genes were analyzed using RT-PCR. RNA was isolated from CPCs as described above. cDNA was prepared using 2 µg of RNA with Superscript III (Life Technologies, Carlsbad, CA). Quantitative real-time polymerase chain reaction (qRT-PCR) was performed using Go-Taq® qPCR Mastermix (Promega, Madison, WI) and the iCycler iQ™5 PCR Thermal Cycler (Bio-Rad, Hercules, CA) following a protocol of 94 °C for 10 min and 45 cycles of 94 °C for 15 s, 46-68 °C (depending on the primer) for 60 s, and 72 °C for 30 s. RT-PCR products were visualized using 1%-2% agarose gel electrophoresis and low mass DNA ladder (Invitrogen, Carlsbad, CA). Primers were designed using the National Center for Biotechnology Information Primer-BLAST program and obtained from Integrated DNA Technologies (Coralville, IA). Primers used in experiments are listed in Table S2.

### Endothelial-like tube formation assay

Matrigel (Trevigen, Gaithersburg, MD) was added to a 96-well plate (50 μL/well) and hardened in a humidified, 5% CO_2_ incubator at 37 °C for 1 h. Cells (20,000 per well) were then incubated for seven hours in EGM-2 media (Lonza Allendale, NJ) before being stained with Calcein AM (Fisher Scientific, Pittsburg, PA) for 30 min. Their ability to form capillary-like networks was measured using an EVOS imaging system (ThermoFisher, Waltham, MA) and quantified with ImageJ (v1.47f, NIH, Bethesda, MD).

### Cell cycle analysis

Aliquots of 250,000 cells were fixed with 70% ethanol overnight, incubated for 60 min with RNAse A (Fisher Scientific, Pittsburg, PA), and stained with propidium iodide, prior to running samples on a MACSquant analyzer (Miltenyi Biotec, Auburn, CA) and analyzing cell cycle progression using the Dean–Jett–Fox model in the cell cycle analysis tool of FlowJo software (Ashland, OR).

### Migration assay

CPCs were trypsinized, counted, and resuspended in starving medium that contained IMDM with GlutaMAX (ThermoFisher Scientific, Waltham, MA), 1% Insulin–Transferrin–Selenium (Life Technologies, Carlsbad, CA), and 0.5% FBS (Thermo Scientific, Waltham, MA). 50,000 CPCs were plated in the top chamber of a 96-well transwell migration assay (Corning, Union City, CA) with 8 μm pores. Standard CPC growth medium supplemented with 100 ng/mL of SDF-1α (Life Technologies, Grand Island, NY) was used in the bottom chamber as a chemoattractant. After 6 h, migrated cells in the bottom chamber were stained using Calcein AM (Fisher Scientific Pittsburg, PA) and quantified using a FLX800 fluorescent plate reader (Bio-Tek, Winooski, VT).

### Statistical analysis

The Shapiro–Wilk test for normality was used to test the normality of data distribution. We then performed a two-tailed, paired *t*-test to compare the mean of all normally distributed data. Non-normally distributed data were compared using a Wilcoxon matched-pairs signed rank text. For cell cycle, migration, and endothelial-like tube formation assay, samples in each group were pooled and either a two-tailed, unpaired *t*-test or Mann–Whitney *U* test was used to compare the mean of normally or non-normally distributed data, respectively. All data are reported as the mean ± the standard error of the mean. Prism 7 version 7.02 (GraphPad, La Jolla, CA) was used for all statistical analyses. *P*-values < 0.05 were assumed to indicate statistical significance.

### Data availability

Gene and microRNA expression data used in RT-PCR, custom array, and microarray experiments have been deposited in NCBI’s Gene Expression Omnibus and is accessible through GEO series accession number GSE110563.^43^ Additional data relating to this publication are available upon request to the corresponding author.

## Electronic supplementary material

Supplemental Files

## References

[1] Fuentes TI (2015). Simulated microgravity exerts an age-dependent effect on the differentiation of cardiovascular progenitors isolated from the human heart. PLoS ONE.

[2] Menasché P (2015). Human embryonic stem cell-derived cardiac progenitors for severe heart failure treatment: first clinical case report. Eur. Heart J..

[3] Mizutani M (2016). Fibrosis of the neonatal mouse heart after cryoinjury is accompanied by Wnt signaling activation and epicardial-to-mesenchymal transition. J. Am. Heart Assoc..

[4] Jha R (2016). Simulated microgravity and 3D culture enhance induction, viability, proliferation and differentiation of cardiac progenitors from human pluripotent stem cells. Sci. Rep..

[5] Meyers VE, Zayzafoon M, Douglas JT, McDonald JM (2005). RhoA and cytoskeletal disruption mediate reduced osteoblastogenesis and enhanced adipogenesis of human mesenchymal stem cells in modeled microgravity. J. Bone Miner. Res..

[6] Louis F, Deroanne C, Nusgens B, Vico L, Guignandon A (2015). RhoGTPases as key players in mammalian cell adaptation to microgravity. Biomed. Res. Int..

[7] Pucéat M, Jaconi M (2005). Ca2+ signalling in cardiogenesis. Cell Calcium.

[8] Bolli R (2011). Cardiac stem cells in patients with ischaemic cardiomyopathy (SCIPIO): initial results of a randomised phase 1 trial. Lancet.

[9] Makkar RR (2012). Intracoronary cardiosphere-derived cells for heart regeneration after myocardial infarction (CADUCEUS): a prospective, randomised phase 1 trial. Lancet.

[10] Gerbin KA, Murry CE (2015). The winding road to regenerating the human heart. Cardiovasc. Pathol..

[11] Hong KU (2014). c-kit+ Cardiac stem cells alleviate post-myocardial infarction left ventricular dysfunction despite poor engraftment and negligible retention in the recipient heart. PLoS ONE.

[12] Fuentes TI (2013). Human neonatal cardiovascular progenitors: unlocking the secret to regenerative ability. PLoS ONE.

[13] Kim TJ (2009). Substrate rigidity regulates Ca2+ oscillation via RhoA pathway in stem cells. J. Cell. Physiol..

[14] Nguyen MTX (2016). Differentiation of human embryonic stem cells to endothelial progenitor cells on laminins in defined and Xeno-free systems. Stem Cell Rep..

[15] Arnaoutova I, George J, Kleinman HK, Benton G (2009). The endothelial cell tube formation assay on basement membrane turns 20: state of the science and the art. Angiogenesis.

[16] Bakker ML, Christoffels VM, Moorman AFM (2010). The cardiac pacemaker and conduction system develops from embryonic myocardium that retains its primitive phenotype. J. Cardiovasc. Pharmacol..

[17] Croquelois A (2008). Control of the adaptive response of the heart to stress via the Notch1 receptor pathway. J. Exp. Med..

[18] Zupanska AK, Denison FC, Ferl RJ, Paul AL (2013). Spaceflight engages heat shock protein and other molecular chaperone genes in tissue culture cells of *Arabidopsis thaliana*. Am. J. Bot..

[19] Versari S, Longinotti G, Barenghi L, Maier JAM, Bradamante S (2013). The challenging environment on board the International Space Station affects endothelial cell function by triggering oxidative stress through thioredoxin interacting protein overexpression: the ESA-SPHINX experiment. FASEB J..

[20] Cubano LA, Lewis ML (2001). Effect of vibrational stress and spaceflight on regulation of heat shock proteins hsp70 and hsp27 in human lymphocytes (Jurkat). J. Leukoc. Biol..

[21] Chen Z, Luo Q, Lin C, Kuang D, Song G (2016). Simulated microgravity inhibits osteogenic differentiation of mesenchymal stem cells via depolymerizing F-actin to impede TAZ nuclear translocation. Sci. Rep..

[22] Chen ZY, Chen F, Cao N, Zhou ZW, Yang HT (2017). miR-142-3p contributes to early cardiac fate decision of embryonic stem cells. Stem Cells Int..

[23] Kang H (2012). Inhibition of microRNA-302 (miR-302) by bone morphogenetic protein 4 (BMP4) facilitates the BMP signaling pathway. J. Biol. Chem..

[24] Porrello ER (2011). MiR-15 family regulates postnatal mitotic arrest of cardiomyocytes. Circ. Res..

[25] Aguirre A (2014). In vivo activation of a conserved microRNA program induces mammalian heart regeneration. Cell Stem Cell.

[26] Jopling C (2010). Zebrafish heart regeneration occurs by cardiomyocyte dedifferentiation and proliferation. Nature.

[27] Baio, J. et al. Spaceflight activates protein kinase c alpha signaling and modifies the developmental stage of human neonatal cardiovascular progenitor cells. *Stem Cells Dev.*10.1089/scd.2017.0263 (2018). 10.1089/scd.2017.0263 29320953

[28] Engler AJ, Sen S, Sweeney HL, Discher DE (2006). Matrix elasticity directs stem cell lineage specification. Cell.

[29] Blaber EA (2015). Microgravity reduces the differentiation and regenerative potential of embryonic stem cells. Stem Cells Dev..

[30] Yuge L (2006). Microgravity potentiates stem cell proliferation while sustaining the capability of differentiation. Stem Cells Dev..

[31] Dai ZQ, Wang R, Ling SK, Wan YM, Li YH (2007). Simulated microgravity inhibits the proliferation and osteogenesis of rat bone marrow mesenchymal stem cells. Cell Prolif..

[32] Zhang S (2015). The effects of spheroid formation of adipose-derived stem cells in a microgravity bioreactor on stemness properties and therapeutic potential. Biomaterials.

[33] Weidgang CE (2013). TBX3 directs cell-fate decision towards mesendoderm. Stem Cell Rep..

[34] Plageman TF, Yutzey KE (2005). T-box genes and heart development: putting the “T” in heart. Dev. Dyn..

[35] Blin G (2010). A purified population of multipotent cardiovascular progenitors derived from primate pluripotent stem cells engrafts in postmyocardial infarcted nonhuman primates. J. Clin. Invest..

[36] Tanaka M, Tickle C (2004). Tbx18 and boundary formation in chick somite and wing development. Dev. Biol..

[37] Hatcher CJ (2001). TBX5 transcription factor regulates cell proliferation during cardiogenesis. Dev. Biol..

[38] Lin SC (2016). Simulated microgravity disrupts cytoskeleton organization and increases apoptosis of rat neural crest stem cells via upregulating CXCR4 expression and RhoA-ROCK1-p38 MAPK-p53 signaling. Stem Cells Dev..

[39] Sharma S (2017). A deep proteome analysis identifies the complete secretome as the functional unit of human cardiac progenitor cells. Circ. Res..

[40] Le TYL (2018). Platelet-derived growth factor receptor-alpha expressing cardiac progenitor cells can be derived from previously cryopreserved human heart samples. Stem Cells Dev..

[41] Smits AM (2009). Human cardiomyocyte progenitor cells differentiate into functional mature cardiomyocytes: an in vitro model for studying human cardiac physiology and pathophysiology. Nat. Protoc..

[42] Vlachos I (2015). DIANA-miRPath v3.0: deciphering microRNA function with experimental support. Nucleic Acids Res..

[43] Edgar R (2002). Gene expression omnibus: NCBI gene expression and hybridization array data repository. Nucleic Acids Res..

