# Supplementary material for: Cardiovascular progenitor cells cultured aboard the International Space Station exhibit altered developmental and functional properties
Source: NPJ Microgravity. 2018 Jul 26;4:13. doi: 10.1038/s41526-018-0048-x (PMC6062551; doi:10.1038/s41526-018-0048-x)
Supplement: Supplementary file 1 — Supplemental Files [file 41526_2018_48_MOESM1_ESM.pdf]

Supplementary Files

Figure S1 presents the representative isotype control staining (gray) for each marker used to detect developmental marker expression in neonatal (blue) and adult (red) CPCs.

Figure S2 outlines the directed differentiation assays for cardiomyocyte, endothelial, and smooth muscle pathway induction (A). Cardiomyocyte (B-C), endothelial cell (D-E), endothelial tube (F), and smooth muscle cell (G-H) induction was screened after 14 days by RT-PCR and flow cytometry. n=3-9 replicates. Data: mean ± SEM, $^{*}p<0.05$, $^{**}p<0.01$, $^{***}p<0.001$.

Figure S3 provides a schematic of the experiment, including how cell culture systems were prepared prior to launch and a timeline of experiments once aboard the ISS.

Figure S4 indicates changes in cardiac (neonatal, A; adult, B) and oxidative (neonatal, C; adult, D) stress response as well as apoptosis activation (neonatal, E; adult, F) in adult and neonatal CPCs flown aboard the ISS for 12 days. n=3 biological replicates. Data: mean ± SEM, $^{*}p<0.05$, $^{**}p<0.01$, $^{***}p<0.001$.

Figure S5 shows the cell count (A) and viability (B) of adult and neonatal CPCs upon return to Earth after 30 days of culture aboard the ISS. n=4 pooled clones measured in technical replicate for cell count and viability results. Data are reported as the mean ± SEM.

Table S1 reports KEGG analysis of targets of significantly altered microRNAs by age.

Table S2 indicates the manufacturer, isotype, concentration, species, clone, catalog number, and lot number of all antibodies used in all experiments.

Table S3 details primer pair sequences (reported 5' to 3') used in RT-PCR experiments as well as the catalog number, manufacturer, and name of microRNA primers that were not originally included in microarray plates.

Figure S1

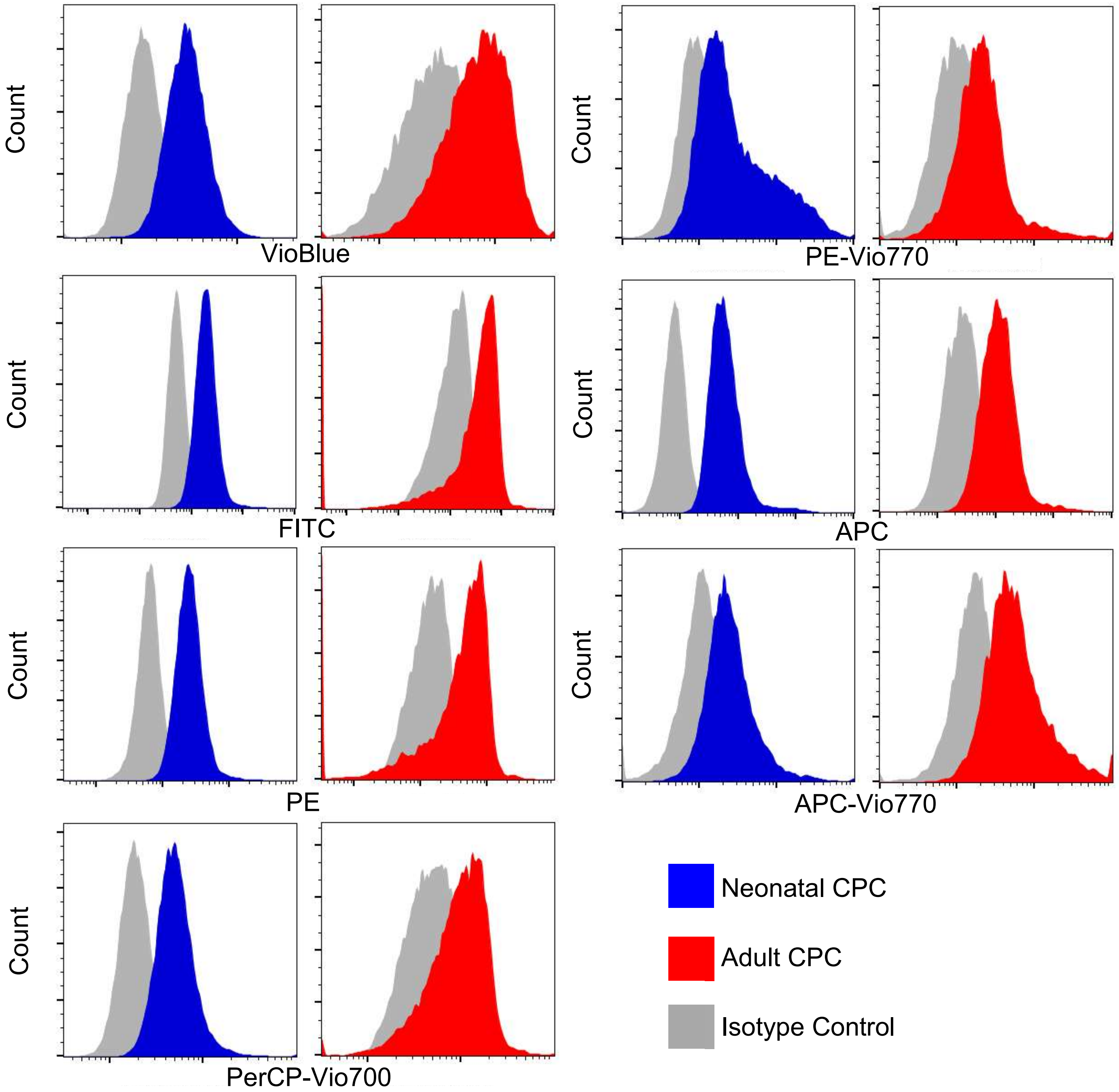
Count
VioBlue
PE-Vio770
FITC
APC
PE
APC-Vio770
PerCP-Vio700
Neonatal CPC
Adult CPC
Isotype Control

Figure S2

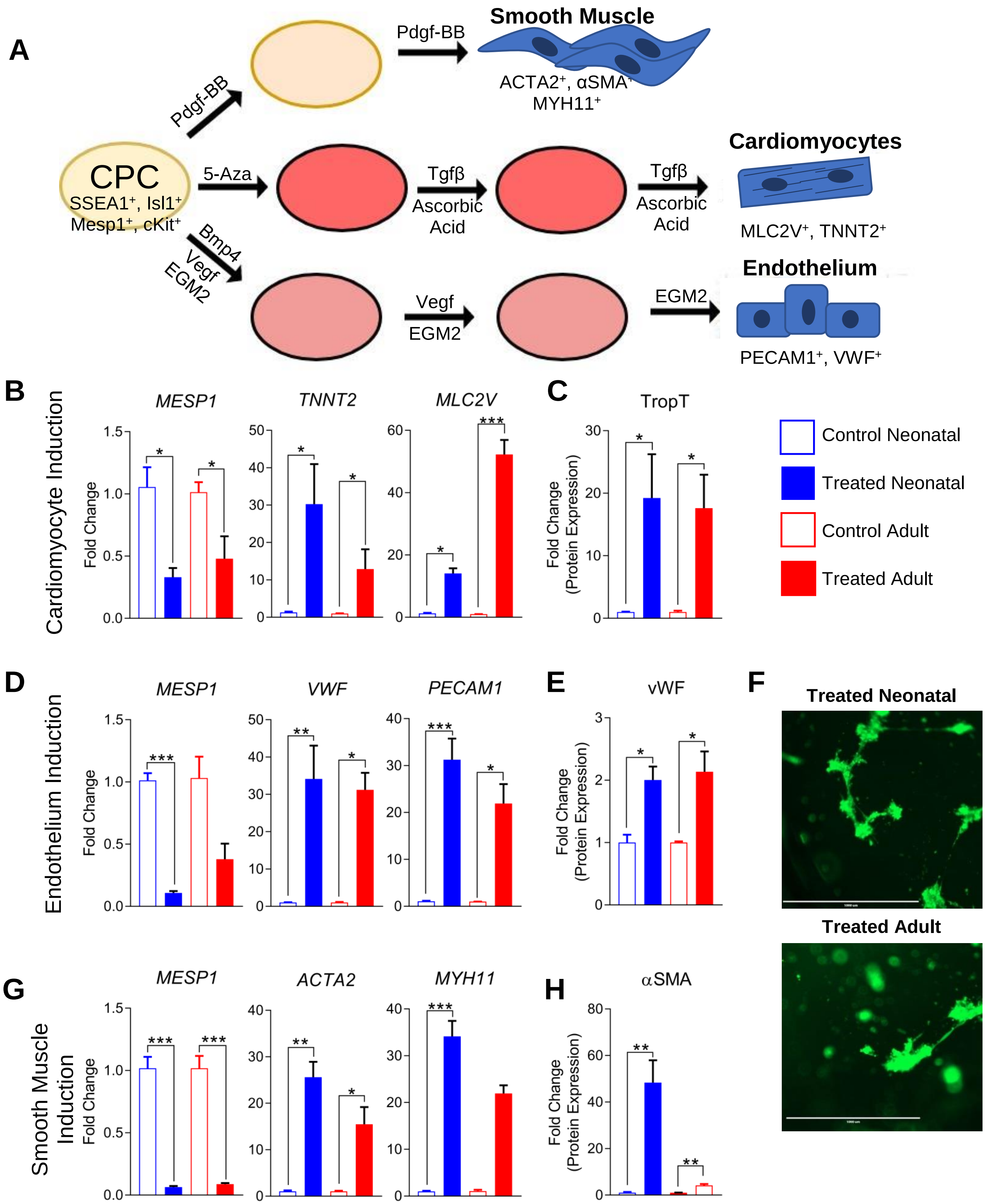
A
Smooth Muscle
Pdgf-BB
Pdgf-BB
ACTA2⁺, αSMA⁺
MYH11⁺
CPC
SSEA1⁺, Isl1⁺
Mesp1⁺, cKit⁺
5-Aza
Tgfβ
Ascorbic Acid
Tgfβ
Ascorbic Acid
Cardiomyocytes
MLC2V⁺, TNNT2⁺
Bmp4
Vegf
EGM2
Vegf
EGM2
EGM2
Endothelium
PECAM1⁺, VWF⁺
B
Cardiomyocyte Induction
MESP1
TNNT2
MLC2V
Fold Change
C
TropT
Fold Change (Protein Expression)
Control Neonatal
Treated Neonatal
Control Adult
Treated Adult
D
Endothelium Induction
MESP1
VWF
PECAM1
E
vWF
F
Treated Neonatal
Treated Adult
G
Smooth Muscle Induction
MESP1
ACTA2
MYH11
H
αSMA

Figure S3

Biocell Seeding

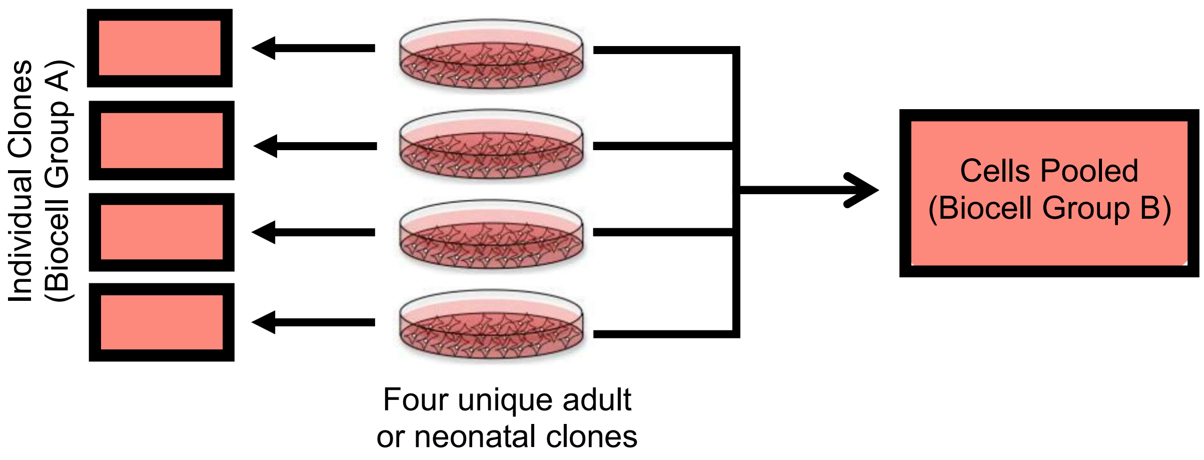

Experiment Timeline

Day 0	Day 3	Day 8	Day 12	Day 16	Day 20
Launch (37ºC & 5% CO_2)	Arrives to ISS<br><br>Media Change (Groups A & B)	Media Change (Groups A & B)	Fixation (Biocell Group A)<br><br>Media Change (Biocell Group B)	Media Change (Biocell Group B)	Media Change (Biocell Group B)

Day 24	Day 27	Day 30	Day 31 – Return to Lab
Media Change (Biocell Group B)	Media Change (Biocell Group B)	Return to Earth (37ºC & 5% CO_2)	Group A<br>RNA Purification<br><br>Group B<br>Endothelial-like Tube Assay<br>Cell Cycle Analysis<br>Migration Assay<br>Protein Isolation

Figure S4

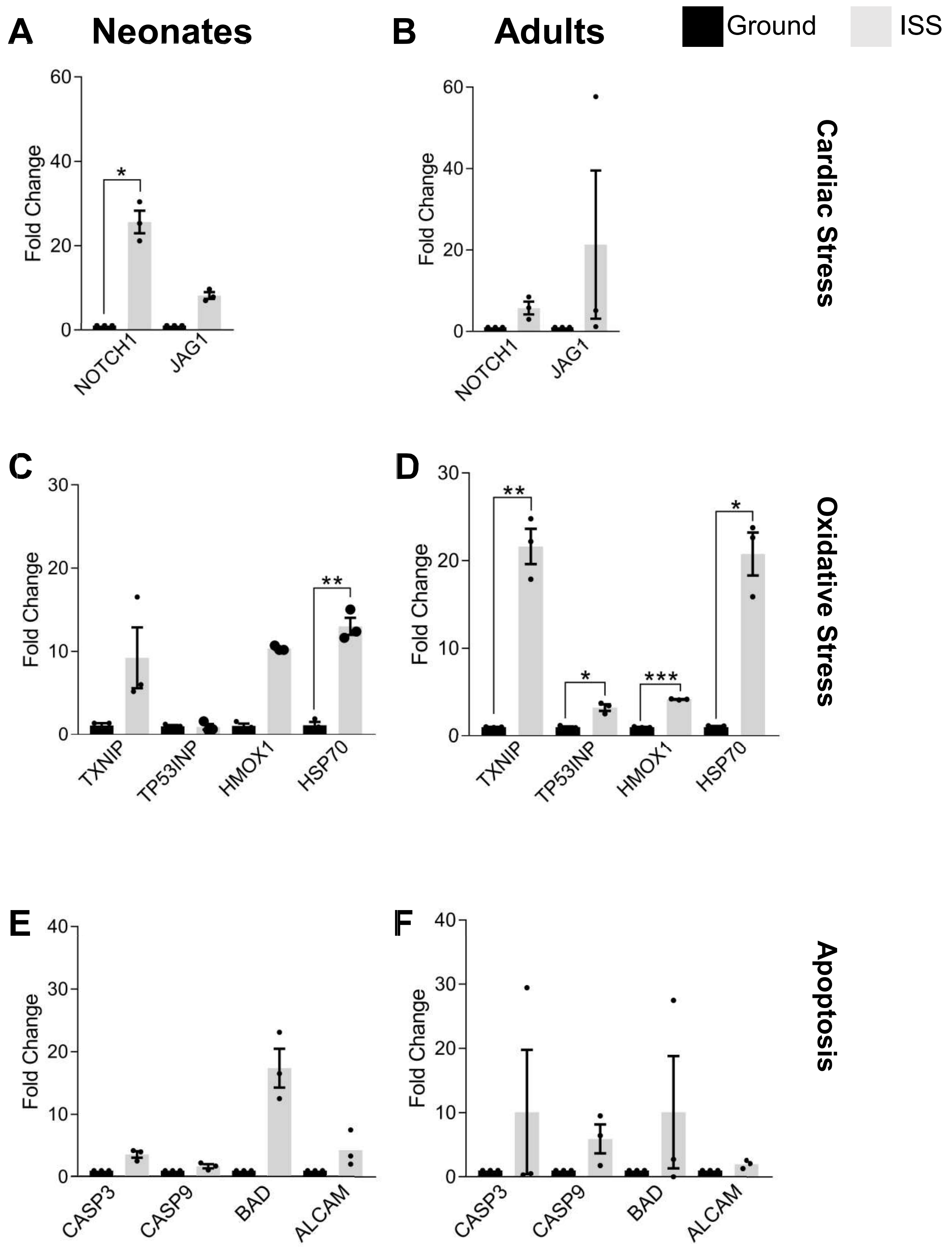

A
Neonates
B
Adults
Ground
ISS
Cardiac Stress
Oxidative Stress
Apoptosis
Fold Change
NOTCH1
JAG1
C
D
TXNIP
TP53INP
HMOX1
HSP70
E
F
CASP3
CASP9
BAD
ALCAM

Figure S5

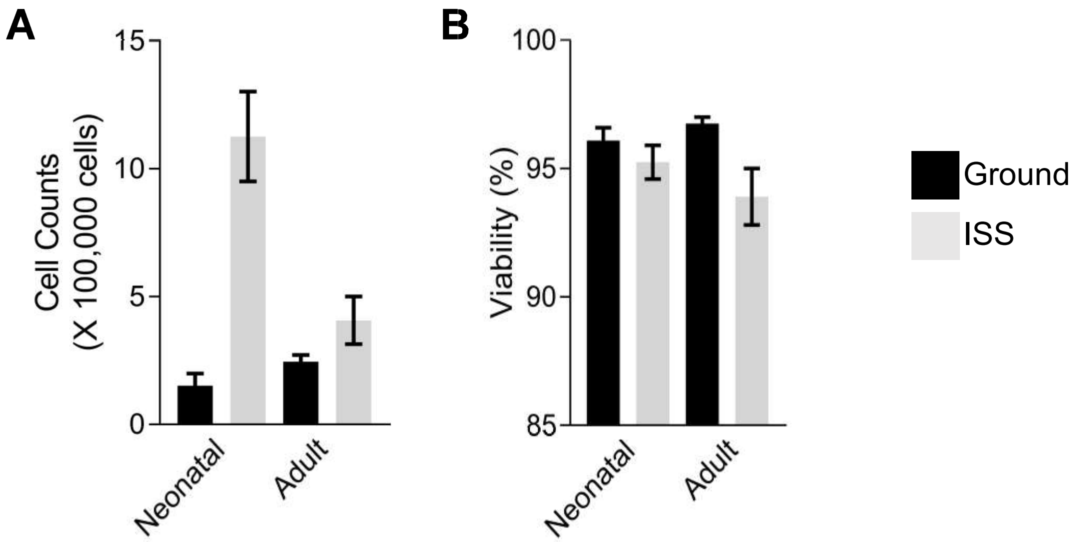
A
15
10
5
0
Cell Counts
(X 100,000 cells)
Neonatal
Adult
B
100
95
90
85
Viability (%)
Neonatal
Adult
Ground
ISS

Table S1

Neonatal CPC KEGG Analysis Results

KEGG pathway	P-Value	Genes	miRNAs
Fatty acid biosynthesis	0	2	1
ECM-receptor interaction	0	14	2
Hippo signaling pathway	1.94E-13	43	3
Cell cycle	1.56E-05	43	3
Lysine degradation	2.80E-05	16	2
Glycosphingolipid biosynthesis - lacto and neolacto series	0.000139519	2	2
p53 signaling pathway	0.002223088	25	3
Protein processing in endoplasmic reticulum	0.02048608	51	2
Endocytosis	0.02304169	52	2
Thyroid hormone signaling pathway	0.04192693	34	2

Adult CPC KEGG Analysis Results

KEGG pathway	P-Value	Genes	miRNAs
Fatty acid biosynthesis	0	4	2
ECM-receptor interaction	0	19	4
Hippo signaling pathway	0	87	8
Lysine degradation	1.11E-16	28	6
Adherens junction	1.26E-10	51	5
Cell cycle	2.97E-09	72	3
TGF-beta signaling pathway	3.00E-08	49	5
Protein processing in endoplasmic reticulum	4.16E-08	99	4
Fatty acid metabolism	3.36E-06	16	2
p53 signaling pathway	2.40E-05	41	4
Endocytosis	0.000157412	76	3
FoxO signaling pathway	0.000502794	51	3
RNA transport	0.0013397	73	2
Thyroid hormone signaling pathway	0.003808595	48	3
Estrogen signaling pathway	0.003832405	29	3
Ubiquitin mediated proteolysis	0.00920295	70	3
Signaling pathways regulating pluripotency of stem cells	0.009475487	56	3
Glycosaminoglycan biosynthesis - chondroitin sulfate / dermatan sulfate	0.01175345	9	2

Table S2

Antibodies used for developmental marker detection

Antibody	Manufacturer	Isotype	Conc.	Species	Clone	Cat. No.	Lot No.
Mesp1-DyLight 405	Novus Biologicals	IgG	0.8 mg/mL	Rabbit	2030B	MAB9219V	CKNQ01-1-050317-V
Isotype-DyLight405	Novus Biologicals	IgG	1.05 mg/mL	Rabbit	NBP2-36463V	NBP2-36463V	31908-050317-V
Viobility 405/520	Miltenyi Biotec	n/a	n/a	n/a	n/a	130-109-814	5170315796
Islet1	Abcam	IgG1	0.83 mg/mL	Mouse	1H9	ab86472	GR273015-3
Fluorescein Labeling Kit	Novus Biologicals	n/a		n/a	10934	707-0030	1BS3384
Isotype-FITC	BioLegend	IgG1 kappa	0.5 mg/mL	Mouse	MOPC-21	400107	B199152
PDGFRα-PE	BioLegend	IgG1 kappa	100 µg/mL	Mouse	16A1	323505	B192368
Isotype-PE	BioLegend	IgG1 kappa	200 µg/mL	Mouse	MOPC-21	400113	B214532
CXCR4-PE/Vio770	Miltenyi Biotec	IgG1	82.5 µg/mL	n/a	REA649	130-109-887	5170503056
REA CtRl-PE/Vio770	Miltenyi Biotec	IgG1	20 µg/mL	n/a	REA293	130-104-616	5170201559
cKit-DyLight650	Novus Biologicals	IgG2B kappa	500 µg/mL	Mouse	2B8	NB100-77477C	B147020-A
Isotype-AlexaFluor 647	R&D Systems	IgG2B	10 µg/mL	Rat	141945	IC013R	AEIU0114121
SSEA1-APC/Vio770	Miltenyi Biotec	IgG1	8.25 µg/mL	n/a	REA321	130-104-992	5161207356
REA Ctrl-APC/Vio770	Miltenyi Biotec	IgG1	30 µg/mL	n/a	REA293	130-104-618	5170201560

Antibodies used for directed differentiation assays

Antibody	Manufacturer	Isotype	Conc.	Species	Clone	Cat. No.	Lot No.
TropT-PE	Miltenyi Biotec	IgG1	10 µg/mL	n/a	REA400	130-106-746	5160426814
Isotype-PE	BD Pharmigen	IgG1 kappa	0.5 mg/mL	Mouse	MOPC-21	556650	19587
vWF-AlexaFluor 657	Novus Biologicals	IgG1 kappa	0.75 mg/mL	Mouse	3E2D10	NBP2-34535AF647	7450-1PABX160519120816-AF647
Isotype-AlexaFluor 647	R&D Systems	IgG2B	10 µg/mL	Rat	141945	IC013R	AEIU0114121
αSMA-PerCP	Novus Biologicals	IgG2a kappa	0.4 mg/mL	Mouse	1A4/asm1	NBP2-34522PCP	59-1PABX170619-090617-PCP
Isotype-PerCP	Miltenyi Biotec	IgG1	55 µg/mL	Mouse	IS5-21F5	130-094-968	5110906188

Table S3

Gene	Forward Sequence (5’ to 3’)	Reverse Sequence (5’ to 3’)
ACTA2	AGCTTTCAGCTTCCCTGAACA	TACAGAGCCCAGAGCCATTG
ACTIN	TTTGAATGATGAGCCTTCGTCCCC	GTCTCAAGTCAGTGTACAGGTAAGC
ATM	GGGCGAGCCGCAAACGCTAA	TTCGGCCCGTCGGAGCAAAC
E2F1	GACCATCAGTACCTGGCCGAGAG	GACGACACCGTCAGCCGAGTG
HGF	CACGAACACAGCTTTTTGCC	TGATCCCAGCGCTGACAAAT
HMOX1	CTCTCGAGCGTCCTCA	ACTATCAGACAATGTTGT
HSP70	TGACCAAGATGAAGGAGATCG	GTCAAAGATGAGCACGTTGC
IGF1	CAGAGCAGATAGAGCCTGCG	CAGGTAACTCGTGCAGAGCA
MESP1	TAGGCCTCAGCGAGGAGAGT	TCCCTTGTCACTTGGGCTCC
MLC2V	GGTGCTGAAGGCTGATTACGTT	TATTGGAACATGGCCTCTGGAT
MYH11	CAAATACGCGGATGAGAGGGA	CTCATGGACGTTCTTGCCCA
NKX2-5	CGCCGCTCCAGTTCATAG	GGTGGAGCTGGAGAAGACAGA
PECAM1	AACGGAAGGCTCCCTTGATG	TAAGAACCGGCAGCTTAGCC
POU5F1	AACCTGGAGTTTGTGCCAGGGTTT	TGAACTTCACCTTCCCTCCAACCA
RAD23A	GTATCGGAGCAGCCGGCCAC	TCCCCAGGGGGCTCGTTCAG
RAD50	CTACGGCTTTGCGTCCCCGG	ACACCAGCTTTCCCCGC
SDF1A/CXCL12	CTACAGATGCCCATGCCGAT	GTGGGTCTAGCGGAAAGTCC
SOX2	AACCAGCGCATGGACAGTTA	GACTTGACCACCGAACCCAT
TERT	AGAGTGTCTGGAGCAAGTTGC	CGTAGTCCATGTTCACAATCG
TNNT2	GTGGGAAGAGGCAGACTGAG	ATAGATGCTCTGCCACAGC
TP53INP1	CACAACAACAAAAGGACTTGGACT	TTGAGCTTCCACTCTGGGAC
TXNIP	TCAGTATTGCAGGGCTTGGC	GTCTCTTGAGTTGGCTGGCT
VEGFA	CAGCGAAAGCGACAGGGGCA	GCTGGAGCACTGTCTGCGCA
VWF	ACACCTGCATTTGCCGAAAC	ATGCGGAGGTCACCTTTCAG
YAP1	TCCCAGATGAACGTCACAGC	TCATGGCAAAACGAGGGTCA

Symbol	MicroRNA	Catalog Number	Manufacturer
hsa-miR-99a-5p	Hs_miR-99a_2	MS00032158	Qiagen
hsa-miR-100-5p	Hs_miR-100	Ms00031234	Qiagen
SNORD72	Hs_SNORD72_11	MS00033719	Qiagen
SNORD96A	Hs_SNORD96A_11	MS00033733	Qiagen
